# Unusual Anterior Choroidal Artery Occlusion in Pediatric Type 1 Diabetes: A Case Report Highlighting Atypical Presentation

**DOI:** 10.7759/cureus.63867

**Published:** 2024-07-04

**Authors:** Roshwanth A, Ramandeep Singh, Ankit Kumar Meena, Cheekatla B Anuhya, Madhu S Gaddigoudar, Arvinder Wander

**Affiliations:** 1 Pediatrics, All India Institute of Medical Sciences, Bathinda, Bathinda, IND; 2 Radiodiagnosis, All India Institute of Medical Sciences, Bathinda, Bathinda, IND; 3 Pediatric Neurology, All India Institute of Medical Sciences, Bathinda, Bathinda, IND

**Keywords:** hemiparesis, pediatric mri, type 1 diabetes, pediatric stroke, anterior choroidal artery

## Abstract

Anterior choroidal artery (AChA) occlusion is a rare but significant vascular event that can lead to severe neurological deficits. Type 1 diabetes mellitus is a known risk factor for various vascular complications, although its association with AChA occlusion in pediatric patients is not commonly seen. A 13-year-old girl, a known case of type 1 diabetes for three years, presented with right-sided headache, visual disturbance in the right eye, and nausea. Magnetic resonance imaging (MRI) of the brain revealed subacute-chronic infarct in the entire left AChA. Internal carotid artery (ICA) stenosis or cardioembolism are the most common causes of complete AChA ischemic strokes. On the other hand, diabetes mellitus, hypertension, and hyperlipidemia usually cause smaller strokes that only affect a part of AChA territory. However, in our case, there was infarct in the entire AChA territory without any cardioembolic risk factor and in the absence of ICA stenosis.

## Introduction

The anterior choroidal artery (AChA), which emerges immediately after the posterior communicating artery (Pcom) origin, is the smallest and most distal branch of the internal carotid artery (ICA) [1]. It can occasionally come from the Pcom, the middle cerebral artery, or the intracerebral carotid bifurcation. It supplies a number of vital brain anatomical structures necessary for vision and motor control. Because of the wide range of variations in its territorial distribution and its strategic and extended region of supply, knowledge of AChA infarct identification is crucial [2,3]. Owing to its blood supply to a wide range of functionally distinct brain structures, its occlusion can lead to variable clinical signs and symptoms. The most common manifestations include hemiparesis or hemiplegia, sensory symptoms affecting the contralateral side, dysarthria, lower facial nerve palsy, visual field disturbances, or even neglect syndrome. The classical triad of contralateral hemiplegia, contralateral hemisensory loss, and contralateral homonymous hemianopia is seen less commonly and is usually seen in complete territorial infarct [4,5]. ICA stenosis or cardio-embolism are the most common causes of complete AChA ischemic strokes. On the other hand, diabetes mellitus, hypertension, and hyperlipidemia usually cause smaller strokes that only affect a part of the AChA territory [5,6]. However, in our case, there was an infarct in the entire AChA territory without any cardioembolic risk factor and in the absence of ICA stenosis.

## Case presentation

The patient was a 13-year-old girl diagnosed with type 1 diabetes two years back. She had been on insulin therapy since then; however, the patient was non-compliant. She reported an episode of right-sided headache four weeks back which lasted for two days for which the patient went to an outside hospital where she was symptomatically treated. Further history showed that the headache was moderate in intensity, and was accompanied by nausea, right-sided visual disturbances, and weakness in the right lower limb. Her visual symptoms persisted along with weakness in her right lower limb. Initial neurological examination showed right upper motor neuron (UMN) facial palsy, right pronator drift, right homonymous hemianopia, and right-sided hemiparesis. Power in the right lower limb was 3/5 and that in the right upper limb was 4/5. 

Her arterial blood pressure was within normal limits. Random blood sugar and glycated hemoglobin (HbA1c) were raised. Among other diagnostic workup renal function tests, lipid profile, ECG, and fundus examination were within the normal limits (Table 1).

**Table 1:** Blood investigations of the patient

Investigation	Result	Unit	Reference Range
White Blood Cells	9.45	x10^3^ /μl	4.00 - 11.00
RBC	4.26	x10^6^ /μl	3.50 - 5.50
Hemoglobin	10.9	gm/dl	11.0 - 16.0
Platelet	196	x10^3^ /μl	150 - 450
Glycated Hemoglobin	12.5	%	Normal <5.7 Prediabetic 5.7-6.4 Diabetic > 6.4
Random Blood Sugar	286	mg/dl	<140
Urea	40	mg/dl	15 - 45
Creatinine	1.0	mg/dl	0.6 - 1.10
Cholesterol	210	mg/dl	<200
Triglyceride	160	mg/dl	<150

**Figure 1:**
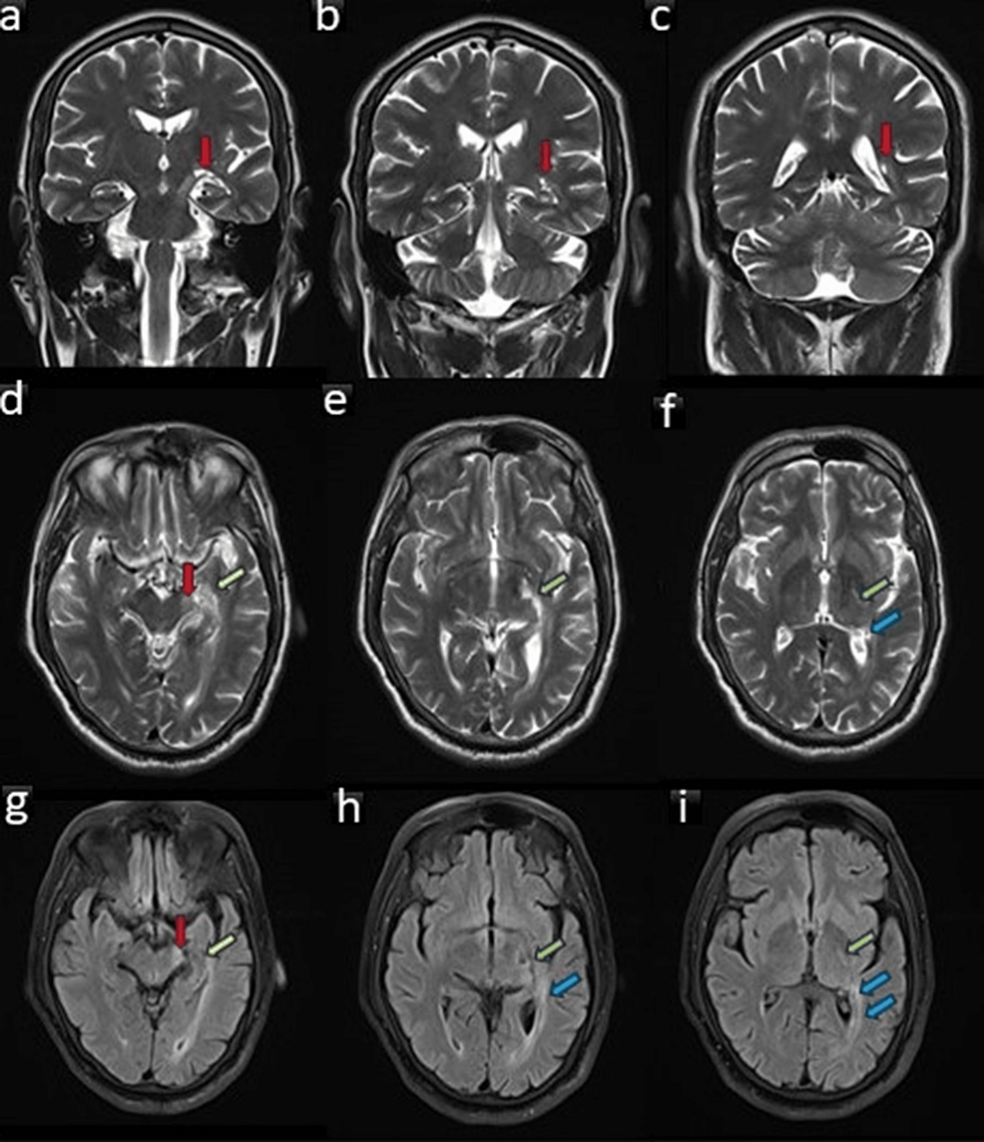
MRI images of the patient showing chronic infarct involving the left anterior choroidal artery territory infarct Coronal T2W brain MRI showing chronic infarct involving the left mesial temporal structures (arrows in a and b) and tail of caudate nucleus (arrow in c). Axial T2W brain MRI at the level of the midbrain (d), hypothalamus (e), and thalamus (f) showing chronic infarct involving the lateral aspect of the left cerebral peduncle (red arrow), mesial temporal structures (yellow arrow), lateral thalamus (green arrows), and optic radiation (blue arrows) on the left side.  FLAIR axial brain MRI images at the levels corresponding to d-f show chronic infarct in the areas as mentioned in images d-f. The infarct involving these areas is typical for anterior choroidal artery territory. FLAIR: fluid-attenuated inversion recovery

A 48-hour inpatient cardiac rhythm monitoring was done, which was not suggestive of abnormalities like atrial fibrillations. Carotid Doppler revealed no significant mural plaques. An MRI brain revealed a subacute-chronic infarct in the left AChA territory, affecting the lateral aspect of the left cerebral peduncle, mesial temporal structures, posterolateral thalamus, and optic radiation on the left side and the tail of the caudate nucleus (Figure 1). There was no diffusion restriction on the MRI to suggest any acute change in the infarct.

Three-dimension (3D) time of flight (TOF) angiography was normal with no evidence of aneurysms/arteriovenous malformations/vascular occlusion. However, the AChA was not demonstrable. Aspirin at a dose of 3 mg/kg was commenced along with physical rehabilitation, leading to a gradual improvement of hemiparesis and visual symptoms. Facial nerve palsy was also partially recovered over a period of three months.

## Discussion

AChA is the small terminal branch of ICA near the Pcom, which goes in a backward and lateral direction towards the inferior horn of the lateral ventricle ending up in the choroidal plexus (intraventricular segment) [3]. AChA territory is tabulated in Table 2.

**Table 2:** Vascular territory of anterior choroidal artery Reference: [3]

Cisternal segment	Intraventricular segment
Posterior limb and retro-lenticular part of the internal capsule	Choroid plexus of the temporal horns of the lateral ventricles
Optic tract	
Optic radiations	
Lateral thalamus including lateral geniculate nucleus	
Lateral cerebral peduncle	
Globus pallidus internus	
Tail of caudate nucleus	
Mesial temporal structures: head of hippocampus and amygdala	

Due to significant anatomical variability and the interchangeability of territories associated with the AchA, its occlusion can lead to a broad spectrum of clinical manifestations. AchA infarcts classically present with a triad of hemiparesis, hemisensory loss, and hemianopia (HHH). Hemiparesis is due to the pyramidal tract's involvement in the posterior limb of the internal capsule, hemisensory loss due to thalamus/thalamocortical fibers, and hemianopia due to the involvement of the retrochiasmatic optic pathway. Frequently, AchA territory infarcts also present as UMN facial nerve palsy and dysarthria. In rare cases, it can also present as nausea and dizziness if it involves posterior circulation but a fact to be noted is that alterations in consciousness and cognition are never observed in AchA territory infarcts [6,7].

The classical triad of HHH with radiological correlation is rarely seen in AchA infarction. This is due to the fact that AchA has rich anastomoses and thus overlaps with the vascular territory of other arteries such as the Pcom, posterior choroidal artery, posterior cerebral artery (PCA), and middle cerebral artery (MCA). Also, the territory supplied by the AchA is relatively small, and infarcts in this area often do not affect all the structures simultaneously. Instead, patients might present with isolated or partial symptoms depending on the specific location and extent of the infarction. AchA infarcts can be categorized radiologically based on their size: small infarcts (diameter <20 mm) and large infarcts (diameter >20 mm) [8,9]. Large AchA strokes, which are uncommon, typically result from significant carotid stenosis or cardioembolic sources. On the other hand, small AchA strokes, often linked to small vessel disease as a result of risk factors such as hypertension, diabetes mellitus, and hyperlipidemia, are more frequently seen as lacunar strokes.

Radiologically, it is crucial to recognize that the entire territory of an AchA infarction cannot be fully visualized on a single axial or coronal image. This limitation arises due to the anatomical location, the extensive area supplied by the artery, and the significant variability in its territorial distribution. Therefore, it is essential to thoroughly examine consecutive stack images from MRI or computed tomography (CT) scans in all three planes (axial, coronal, and sagittal) to accurately determine the extent of the infarction. Additionally, due to its strategic supply, an AchA infarct can be easily mistaken for an MCA, PCA, or MCA-PCA watershed infarct [10]. The AChA may not be seen in up to 5-30% of cases on angiogram (as in the index case) due to its small diameter or because it may be obscured by overlapping vessels [11].

Our case is unique in terms of clinical presentation because headache was the predominant and the presenting symptom. Also, there was involvement of almost the entire AchA territory on the MRI brain in the absence of any cardioembolic source or ICA occlusion. To the best of our knowledge, complete AchA territorial infarct in a child with Type 1 diabetes mellitus has never been reported before.

The pathologies involving the AChA range from infarction, aneurysm, Moyamoya disease, brain arteriovenous malformations (AVM), or arteriovenous fistula (AVF), to tumors. Isolated AChA infarcts are rare, with a prevalence ranging from 2-11% [5].

Logically, we may think that small vessel pathology like vasculitis is more commonly involved in AchA infarcts but many studies reported that the large vessel infarcts resulting from atherosclerosis, and thromboembolism account for most cases of AchA infarcts. The prevailing stroke mechanism is a small-vessel occlusive disease, primarily identified in hypertensive and diabetic patients.

Therefore, a holistic diagnostic stroke workup is essential for all cases of AchA territory strokes rather than focusing only on small vessel pathologies which includes CT/MR angiography, MRI, 2D-echocardiography, 24-hour rhythm Holter (atrial fibrillation), continuous blood pressure monitoring, and carotid doppler.

The current standard treatment of care for AchA territory infarcts includes analyzing the cause of the infarct and working on it. Drugs given may include dual antiplatelet therapy (DAPT), which includes aspirin and clopidogrel, anticoagulation drugs for venous thromboembolism like low molecular weight heparin (Enoxaparin), antihypertensives (amlodipine), and statins (for dyslipidemia). Thrombolytic drugs are also tried for AchA territory stroke but there is variable symptomatic improvement seen in cases of AchA strokes [10].

## Conclusions

This case highlights the rare occurrence of complete AChA occlusion in a pediatric patient with type 1 diabetes mellitus, emphasizing the importance of considering atypical etiologies in stroke presentations. Further research is needed to elucidate the mechanisms underlying such occurrences and guide appropriate management strategies.
